# Over One-Year Long-Term Laboratory Tests of pH Electrodes in Terms of Industrial Applications Checking Stabilities of Their Parameters and Their Influence on Uncertainties of Measurements

**DOI:** 10.3390/s18124102

**Published:** 2018-11-23

**Authors:** Alicja Wiora, Józef Wiora

**Affiliations:** Institute of Automatic Control, Silesian University of Technology, ul. Akademicka 16, 44-100 Gliwice, Poland; alicja.wiora@polsl.pl

**Keywords:** pH measurement, glass electrode, long-term stability, prediction, hysteresis, measurement uncertainty

## Abstract

Glass pH electrodes are still successfully applied in the chemical and environmental industry. During their long-term use, periodic calibration is required to maintain the required accuracy of measurements because the parameters of the electrodes change over time. This work presents an aging of 11 pH electrodes within approximately 600 days in tap water. During this period, potentials of all electrodes in five buffer solutions were measured 44 times. This allowed determining the aging models of the electrodes. Models in other mediums might be different. Changes in slope, standard potential, hysteresis, and linearity of the characteristics were the objects of observations. A method for predicting electrode parameters is proposed. Furthermore, the procedure for estimating the uncertainty of pH measurements considering the aging of the electrodes is described. As a result of this work, a model of the aging process of pH electrodes in tap water can be constructed and subsequently, the measurement accuracy in the periods between calibrations can be improved.

## 1. Introduction

Measurements of pH values belong to the most frequent chemical assays conducted in industrial application. They provide an indication regarding how the production process is carried out. Often, government regulations require measurements of the value to satisfy environmental protection. Among the available methods, potentiometric measurements benefit from their simplicity and the ease of signal transmission. The disadvantages include the need for periodic calibration, which often requires manual removal of the electrode from the measuring system [1,2,3,4,5]. For the presentation of results, the potentiometric sensors require an uncomplicated setup and measuring transducer. Maintenance and laboratory calibration require moderate efforts. In situ measurements allow the provision of real-time information for examined quantity. It makes them suitable for industrial and field application. On the other hand, they are less precise compared to other analytical techniques [6]. Therefore, knowledge regarding the aging process is highly significant. Its mathematical modeling allows reducing the frequency of servicing or increasing accuracy while the periods of calibration are maintained.

Each measurement result should contain information not only regarding its value but also its quality. Most instruments provide only current indications while the measurement values emerge after information processing such as filtering, averaging, and correction of systematic effects. The quality of the measurement is provided less frequently. Currently, it is recommended to be presented as measurement uncertainty evaluated according to the guide for the expression of uncertainty in measurement (GUM) [7] or many other manuals based on the former (e.g., [8,9,10]). This work focused on building such a model of the aging process of glass pH electrode, which ensures the lowest possible measurement uncertainty during its prolonged use.

## 2. Potentiometric Principles

Potentiometric measurement of pH value is realized using a pH electrode which works together with a reference electrode. The two parts are often constructed as one sensor called the combination electrode. On its output, a voltage arises which is dependent on the concentration of hydrogen ions H^+^ or, more strictly, hydronium ions H_3_O^+^. In the chemical nomenclature, the voltage is known as electrode potential because the potential of the reference electrode is nearly constant and independent of ion concentration. Omitting the so-called liquid junction potential arising between the reference electrode and the measured solution, the electrode potential *E* is modeled by the Nernst equation [11]

(1) $$E=E^{\circ }+S\log\left(a_{H+}\right),$$

where $E^{\circ }$ is the standard potential, *S* is the Nernstian slope, and $a_{H+}$ is the activity of $H^{+}$ ions. According to IUPAC, the pH value is defined as

(2) $$\mathrm{pH}=-\log\left(a_{H+}\right).$$

Therefore, the potential is dependent on the pH according to

(3) $$E=E^{\circ }-S\cdot \mathrm{pH}.$$

Parameters of the equation change with time due to an aging process. Therefore, calibrations should be performed periodically, and more frequently when higher precision is required. The potential is measured by a voltmeter called the pH-meter or ion-meter. It should provide measurements without current flow; thus, the pH-meter requires extremely high input impedance and very low bias current [12,13]. The meters typically have cable connections. In environmental measurements, wireless communication such as ZigBee are preferred, due to their low power consumption [14,15,16]. On mains power, a Wi-Fi module could be installed with other facilities, such as a web server [17].

Stability of the reference electrode directly influences the stability of pH measurement. Contemporary reference electrodes are Ag/AgCl type with aqueous or hydrogel-trapped KCl solutions, with solid melt of the metal salt or all-solid. Conventional reference electrodes have a few μV/day potential drift; it is 1000 times higher in other constructions. Quasi-reference electrodes that are built from inert noble metals, metal salts, or metal oxides have stability dependent on the measured solution [18,19].

Literature describes long-term measurements conducted with glass pH electrodes. We found 500-day recordings of potential changes in four buffer solutions. However, the phenomenon is not modeled [20]. The other is a 24 h test of small combined pH electrodes used in clinical applications [21]. A 70-day test of polymer-based pH electrodes with comparable performances to glass electrodes exhibits some potential scatters. However, the authors did not present the time dependencies [22].

Metal-oxide pH electrodes are robust, inert, miniaturizable, mechanically strong, and effective in aggressive environments at a wide range of temperatures and pressure. However, they are sensitive to oxidizing/reducing agents [23,24,25]. Authors of an eight-day test of metallic tungsten and tungsten oxide electrodes ensured their stability, but did not provide any quantitative description [26]. Others described a ruthenium oxide (RuOx) sensor: when the electrode is stored in liquid, its 0.8 mV/h drift is shorter than when stored in air [23]. A 1200-h test of antimony oxide (Sb_2_O_3_) electrodes displays a 4 mV potential change. The researchers claimed this to be a result of the evaporation of water from the sample [24]. A ruthenium oxide (RuO_2_) electrode drifts only through 10 min at 50 ${}^{\circ }$C. The drift is not described quantitatively [25]. The other RuO_2_ electrode exhibits hysteresis, also called memory effect, assessed to 10 mV. An initial drift of approximately 79 mV is observed. The authors did not notice any difference in the slope and the standard potential between 24 and 48 h [27]. The potential of an iridium oxide (IrOx) electrode measured before and after the 2.5-year period “just overlapped each other”—the magnitude of differences is unknown [28].

Ion-sensitive field-effect transistors (IS-FETs) require much less complicated electronics than conventional glass electrodes. Good IS-FETs achieve a long-term stability of a few mV/h typically; some even reach 59 mV/h, which is approximately 1 pH/h [29]. It makes them unsuitable for automatic or remote measurements. However, they are used for 21-h acidity monitoring in the upper gastrointestinal tract. To improve stability, the electrodes are immersed in pH solutions for at least six hours prior to use. An initial drift of up to 15 mV/h is observed in the first four hours. Afterwards, however, the drift reduces to less than 15 mV/day [30]. Another work confirms the initially higher drift which decreases with time. In a 600-day test, the slope of an IS-FET stored in deionized water remains stable within 55±1 mV/pH, and its standard potential drifts linearly with 0.35 mV/day.

Long-term tests may consider materials such as concretes. To measure their resistance to chloride penetration, the chloride concentration in a sample is tested in an apparatus on Days 28, 90, 140, 365, and 1095. Properties of sensors are assumed unchanged [31]. In other studies, over 550-day tests of concretes include pH measurements [32]. Elaborated procedures describe pH measurements of fresh and hardened concrete. Some of them use potentiometric pH electrodes [33].

Despite long-term tests conducted by several authors, there is a lack of mathematical modeling involving the aging process that would improve metrological properties of the most popular commercially available glass pH electrodes. Therefore, we decided to examine several popular types of electrodes available in Poland. During the nearly 600-day test, the attention was focused on the drift of calibration parameters. Based on the collected data, the typical behavior of the electrodes was determined. It allowed for the prediction of the parameters and improves the results of measurements without very frequent calibrations.

## 3. Materials and Methods

Eleven pH combination electrodes were investigated. They are listed in Table 1. Only two of them were of the same type and age. The E12 electrode was purchased from Elmetron, Zabrze, Poland and others from Hydromet, Gliwice, Poland. Electrodes E01–E12 were not used before investigations, while E13 was used by students for approximately two years.

The buffer solutions were stored at room temperature in 100 mL bottles filled three-quarters and were immersed in a 25 ${}^{\circ }$C water bath before measurements. The measurement procedure for each of the electrodes was as follows: The investigated electrode was connected to the ORION Model 930 Ionalyzer (Orion Research, Cambridge, MA, USA). Its membrane was rinsed manually for 5 s in distilled water, immersed in the bottle with buffer solution, and stirred manually for 5 s. Subsequently, it was attached to a stand and held in the buffer solution without stirring. Potential indications were observed in the following moments of time: 0:40, 1:00, 1:15, 1:30, 1:45, 2:00, 2:10, 2:20, 2:30, 2:40, 2:50, 3:00, 3:10, 3:20, 3:30, 3:40, 3:50, 4:00, 4:10, 4:20, 4:30, 4:40, 4:50, and 5:00 min. If there was no change in the electrode potential at the least significant digit, i.e., 0.1 V, between two subsequent readings, then stable state was assumed and the potential was noted. If the stable state was not achieved, then the indication after 5 min counted from immersing in the solution was noted. After the reading was recorded, the electrode was stored together with other electrodes in a beaker with tap water that was refreshed after each measurement series. Parameters of the tap water, according to information from the local water supply network (http://pwik.gliwice.pl/tabele-z-parametrami.html) in the period of experiments ranged as follows. Acidity: 7.5–7.7 pH; conductivity at 25 ${}^{\circ }$C: 682–904 µS/cm; hardness: 332–356 mg/L CaCO_3_; concentrations of the following particles—${\mathrm{NH}}_{4}^{+}$: <0.064 mg/L; ${\mathrm{NO}}_{3}^{-}$: 1.5–16.3 mg/L; ${\mathrm{NO}}_{2}^{-}$: <0.05 mg/L; ${\mathrm{Fe}}_{3}^{+}$: 9.9–46.7 µg/L; Mn: 1.9–5.14 µg/L;

We performed 44 measurement series. Initially, the experiments were conducted every day; later, they were held less often; and finally they were conducted approximately every six weeks. Each measurement series consists of the testing of every electrode in five pH buffer solutions obtained from the Avantor Performance Materials Poland S.A. (Gliwice, Poland). The pH values were 2, 4, 7, 9, and 10 with a tolerance of ±0.05 pH. To test hysteresis, the order of measurements was switched. This means that, on the first day, the order was from pH 2 to pH 10, and, in the subsequent series, it was from pH 10 to pH 2. The order of the electrodes was also switched every two measurement series. This means that, in the first two series, electrodes were tested beginning from E01 to E13 and, in the next two series, from E13 to E01.

Buffer solutions were poured from 1 L bottles into 100 mL plastic working bottles. Measurements were performed in the working bottles. The rest of the solutions in 1 L bottles was stored. Due to the cost of the experiments, buffer solutions were used in several series. The working bottles were opened before the measurement series and stayed opened during all tests and were closed after the series. Electrodes were plugged in subsequent buffers without sealing, which led to contamination of the buffers, although the electrodes were rinsed in water. After several series, the potentials of electrode E06 in the working bottles were compared with potentials in the 1 L bottle. The obtained potential differences were used for calculating the corrections of pH in working bottles by applying the Nernst equation and the electrode slope determined earlier. pH values of solutions in working bottles in all prior series after the last comparison were corrected assuming a linear change in pH with the number of series. If the difference was larger than 10 mV, the buffer solution in the working bottle was replaced with that in the stored bottle.

## 4. Results and Discussion

### 4.1. The Change of Potentials

**Figure 1:**
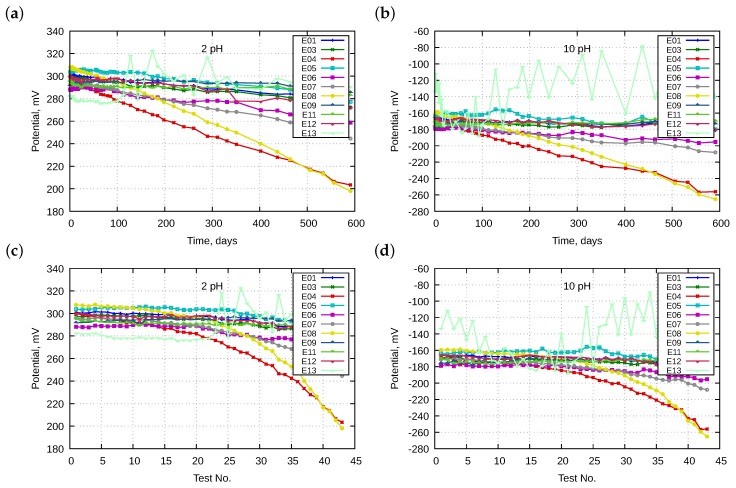
Change of potentials of electrodes E01–E13 in the same buffer solutions: (**a**,**b**) as a function of time in pH 2 and 10, respectively; and (**c**,**d**) as a function of the test number.

The analysis of drift of potential over time started with a comparison of the potential of all electrodes immersed in the five buffer solutions. Examples of characteristics are presented in Figure 1.

Potentials of all electrodes, except for the oldest E13, in all the buffer solutions tended to lower their values with over time. The aging process was dependent on the electrode type. At the beginning of the investigations, the potential span between electrodes was approximately 25 mV, while at the end was wider at approximately 100 mV. Electrodes E04 and E08 displayed the most significant drift. The panel a and b of Figure 1 present potential as a function of time, whereas panels c and d indicate it as a function of the test number. We conclude that the drifts exhibited linear time-dependence; the characteristics were definitely less linear in the second case because the intervals between the tests were not the same. Initially, the tests were performed more often. This means that the drift was caused more by the lifetime of the electrodes than by the exploitation work. Therefore, further dependencies were only sought as functions of the lifetime. 

We observed different rates dependent on electrodes which proved that the drifts were caused by aging processes. Therefore, the changes in pH of the working buffer solutions have a significantly lower impact. Additionally, we compared periodically pH in working bottles with these in 1 L bottles which were not used in the meantime. The calculated differences led to pH corrections in working bottles.

**Figure 2:**
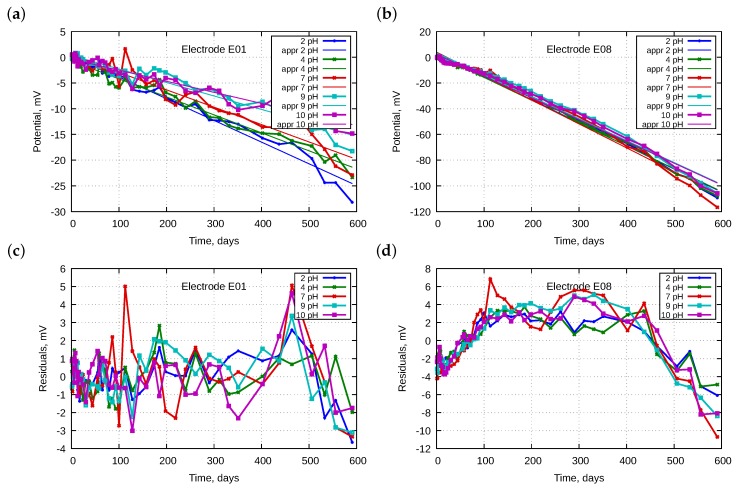
Examples of electrode potential changes: (**a**,**b**) standardized potentials with fitted straight lines as a function of time for electrodes E01 and E08, respectively; and (**c**,**d**) with corresponding residuals.

The linearity of the drift of each electrode was tested by plotting the residuals. Figure 2 presents examples of such plots. The figure should be singular, was plural consist of pairs of panels where the upper ones present potentials and the lower ones present residuals. To facilitate the visual comparison, potentials measured in all buffer solutions were shifted to obtain zero volts at the beginning of investigations. We also added approximation lines. Residuals were calculated as the differences between measured values and the calculated approximation function. Nearly all electrodes exhibited residuals randomly distributed around 0; therefore, the linear approximation is justified. Only E08 showed a parabolic character. Therefore, its drift model should be a higher order function. The old E13 electrode exhibited strong hysteresis, increasing with its lifetime. This is discussed in more detail in Section 4.6.

### 4.2. Drifts of the Parameters of the Nernst Equation

For typical users, it is not only the potential changes in time that are instrumental, but also the changes of the electrode parameters. We estimated the parameters after each measurement series performed in one day using ordinary least squares and fitting the Nernst Equation (3). In this way, two parameters were obtained: electrode slope *S* and standard potential $E^{\circ }$.

**Figure 3:**
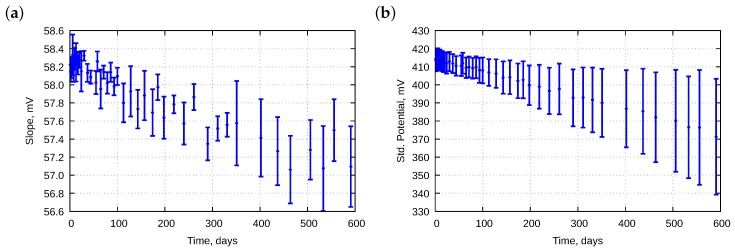
Averaged change of (**a**) slopes and (**b**) standard potentials of electrodes E01–E12 in time. Bars mean standard deviations.

Figure 3 presents their variability in time. The figure regards electrodes E01–E12; electrode E13 is omitted because its parameters significantly differ from others. We see that the values of the parameters lower with time, whereas the deviations of the parameters increase with time. Deeper analyses are provided below. 

#### 4.2.1. Slope

**Figure 4:**
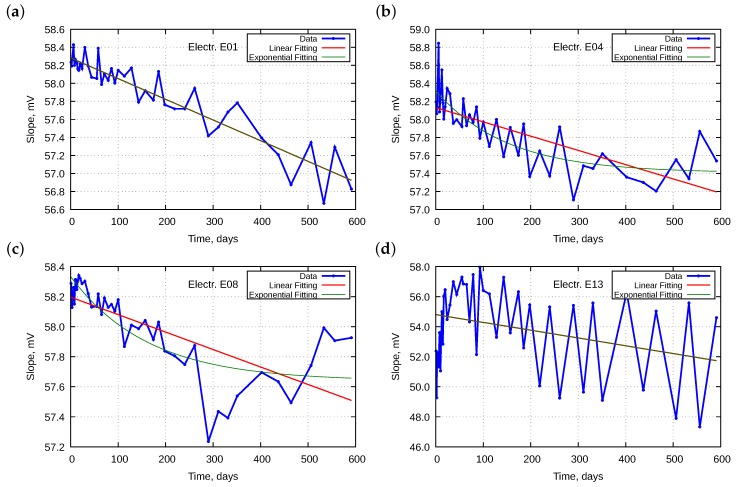
Examples of changes of electrode slopes in time for electrodes: (**a**) E01; (**b**) E04; (**c**) E08; and (**d**) E13.

Figure 4 presents changes of the electrode slopes *S* as a function of their lifetime *t*. Additionally, we added two approximation lines which minimize the sum of the squares of residuals (SSR). The first one shows the linear fitting of the following model:

(4) $$S=S_{0}+\Delta S\cdot t,$$

where $S_{0}$ is the initial value of the slope, ΔS is its daily change, and *t* is the lifetime. The second line arose as an exponential fitting to the following model:

(5) $$S=S_{\infty }+\left(S_{0}-S_{\infty }\right)\exp\left(-t/T_{S}\right),$$

where $S_{\infty }$ is the final value at a hypothetical infinite lifetime and $T_{S}$ is the time constant. We observed the initial daily change $\Delta S_{0}$ calculated as

(6) $$\Delta S_{0}=\frac{\partial S}{\partial t}{|}_{t=0}=-\frac{1}{T_{S}}\left(S_{0}-S_{\infty }\right)\exp\left(-t/T_{S}\right){|}_{t=0}=-\frac{1}{T_{S}}\left(S_{0}-S_{\infty }\right).$$

Table 2 collects the obtained approximation parameters.

The calculated SSR indicates how well the lines fit the experimental data.

Although electrodes E01–E09 were new and all measurements were performed at 25 ${}^{\circ }$C, their initial slopes differed from the theoretical value of 59.16 mV. Obtained initial slopes ranged from 58.06 to 58.39 mV for linear approximation and from 58.19 to 58.39 mV for exponential approximations. The SSR obtained for the oldest electrode E13 was significantly worse than that for other electrodes. The hysteresis effect shown in Figure 4d and described broadly in Section 4.6 dominated for the electrode. Consideration of its other parameters is meaningless. For the linear approximation, other electrodes exhibited comparable daily changes of slope in the range from −1.1 to −3.1 µV/day. The SSR values ranged from 0.47 to 3.68 ${\mathrm{mV}}^{2}$.

Application of the exponential approximation primarily improves the SSR values that are larger. For four cases (E01, E03, E06, and E07), the found time constant was extremely large; in our time horizon, therefore, the exponential function became a straight line. For other cases, the non-linear approximation improved the SSR. We cannot, however, theoretically justify the extrapolated final values of the slopes, which were relatively broadly spread from 55.98 to 57.70 mV, and of the time constants, which ranged from 116 to 745 days. The initial daily changes were not lower than the daily changes obtained from linear approximation and ranged from −2.1 to −8.2 µV/day. All cases with absolute values of daily change ΔS lower than 2 µV/day increased their initial daily change $\Delta S_{0}$. This implies that new electrodes exhibit faster slope drift than older ones.

#### 4.2.2. Standard Potential

**Figure 5:**
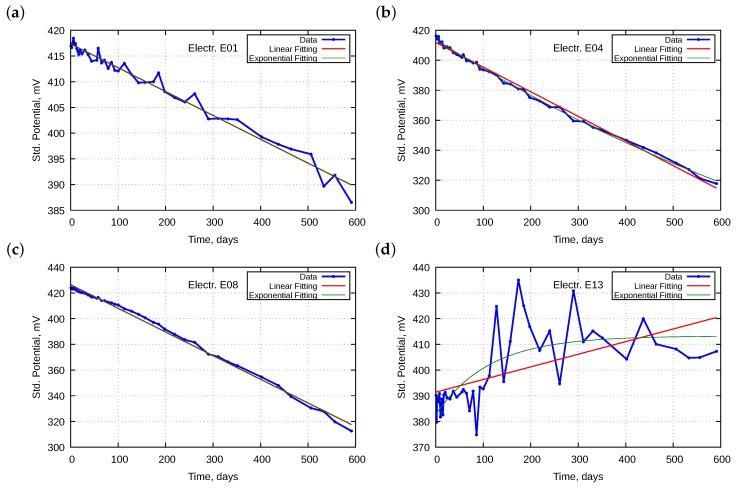
Examples of changes of electrode standard potentials in time for electrodes: (**a**) E01; (**b**) E04; (**c**) E08; and (**d**) E13.

We conducted similar analyses on those performed for the slope drift and also for the drift of the standard potential. Figure 5 presents changes of the standard potentials $E^{\circ }$ of the pH electrodes as a function of time *t*. Additionally, we plotted two approximation functions, linear and exponential, using the following relationships:

(7) $$E^{\circ }=E_{0}^{\circ }+\Delta E^{\circ }\cdot t,$$

(8) $$E^{\circ }=E_{\infty }^{\circ }+\left(E_{0}^{\circ }-E_{\infty }^{\circ }\right)\exp\left(-t/T_{E^{\circ }}\right),$$

where $E_{0}^{\circ }$ is the initial value of the standard potential; $\Delta E^{\circ }$ is its daily change; $E_{\infty }^{\circ }$ is the final value at a hypothetical infinite lifetime; and $T_{E^{\circ }}$ is the time constant. The initial daily change of the standard potential $\Delta E_{0}^{\circ }$ was calculated from:

(9) $$\Delta E_{0}^{\circ }=\frac{\partial E^{\circ }}{\partial t}{|}_{t=0}=-\frac{1}{T_{E^{\circ }}}\left(E_{0}^{\circ }-E_{\infty }^{\circ }\right).$$

Table 3 includes the calculated parameters.

Similar to the slope analysis, E13 had at least one order greater SSR factors. This means that it worked in an unrepeatable way; therefore, further analysis does not include it.

The initial standard potential ranged from approximately 406.5 to 426.4 mV and its span was approximately 20 mV. After 600 days, it ranged from 313.3 to 403.6 mV and the span was 90 mV. This implies that the aging process does not proceed at the same rate in all electrodes. All electrodes, except the oldest E13, exhibited negative standard potential drift. The absolute value of the drift was in a very wide range from 11 to 184 µV/day. Applying exponential approximation, the initial daily change reached even 202 µV/day for E04. Only for this electrode, the exponential approximation significantly improved the calculated SSR factors. The obtained time constant was the lowest at 1196 days but was still very high. Others time constants were even larger; therefore, the linear approximation was good enough for modeling the phenomenon.

### 4.3. Time-Independent pH Value

Parameters $E^{\circ }$ and *S* were correlated in the Nernst equation [34]. Using the linear aging model, we could find such a pH value for which electrode potential remains constant and independent in its lifetime. The pH value, e.g., ${\mathrm{pH}}_{\mathrm{indep}.}$, was obtained by combining Equation (3) with Equations (4) and (7):

(10) $$E\left(t\right)=E_{0}^{\circ }+\Delta E^{\circ }\cdot t-\left(S_{0}+\Delta S\cdot t\right)\cdot \mathrm{pH}.$$

The potential is time-independent if $\frac{\partial E(t)}{\partial t}=0$. Thus,

(11) $${\mathrm{pH}}_{\mathrm{indep}.}=\frac{\Delta S}{\Delta E^{\circ }}.$$

Table 4 collects the calculated ${\mathrm{pH}}_{\mathrm{indep}.}$.

The obtained values were spread very broadly and exceeded the useful pH range. Only electrodes E09 and E11 offered a ${\mathrm{pH}}_{\mathrm{indep}.}$ value that was possible to produce. Therefore, we could not predict such a pH value for which the electrode potential remains unchanged over its lifetime.

### 4.4. Prediction of Electrode Parameters

**Figure 6:**
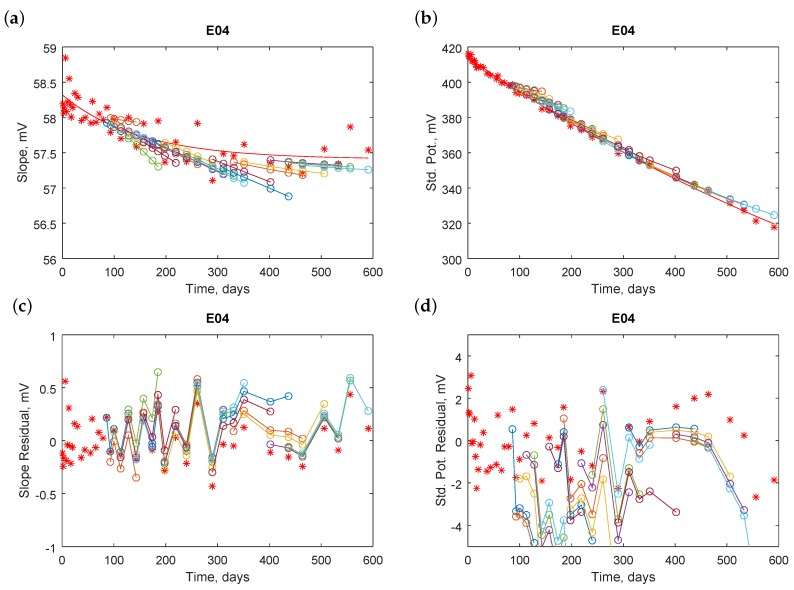
Illustration of performance of predictions for electrode E04: (**a**) electrode slope; (**b**) electrode standard potential; (**c**) slope residuals; and (**d**) standard potential residuals. Red stars are experimentally obtained electrode parameters (upper panels) or residuals (bottom panels), the red line is the off-line calculated approximation function, and color circles with lines are predicted values. Each line in a particular color implies that the same prediction parameters are used for predicting future values based on the past data.

Knowing a model for the changes of electrode parameters during the lifetime, we could predict future potentials. We tested efficiency of the predictions for every electrode. Starting from the tenth calibration, we calculated an aging model fitted to an exponential function. Electrode parameters were predicted for the next 1, 2, …, 5 measurements, respectively. We compared the predicted parameters with parameters obtained experimentally. Figure 6 presents an example (not the best of all collected) of electrode E04.

The predicted model for the slope (Figure 6a,c) is more linear at the beginning than that calculated for all the data. Therefore, predictions for several steps ahead differ significantly from experimental values. Table 5 presents the calculated standard deviations of the differences, which are a measure of the prediction quality.

The values could be treated as on-line quality factors because the predicted slopes may be calculated during electrode exploitation. The column called “off-line” includes standard deviations calculated after the nearly 600-day acquisition; however, we could not apply slopes obtained in this manner for calculations of measured pH during the exploitation. The quality factors obtained for predictions were slightly worse than those obtained after all experiments. Usually, if more future values were predicted, the factors were worse.

Similar plots are prepared for standard potentials. An example is presented in Figure 6b,d. The predictions seem quite promising but results from a fast drop of the standard potential with time. The predictions for one or two steps ahead are reasonably good. The quality can be assessed quantitatively, as it is presented on the right hand side of Table 5.

The practice of two calibrations conducted before and after a measurement to decrease measurement errors is well known in laboratory experiments. Such an approach would be, however, impossible in the case of an industrial application with a long pause between the calibrations. Therefore, the prediction of the change of calibration parameters until the next calibration based on observed history is a legitimate alternative to relying on the results of the last calibration. It allows for an improvement of measurement results in the time between calibrations.

### 4.5. The Change of Electrode Linearity

**Figure 7:**
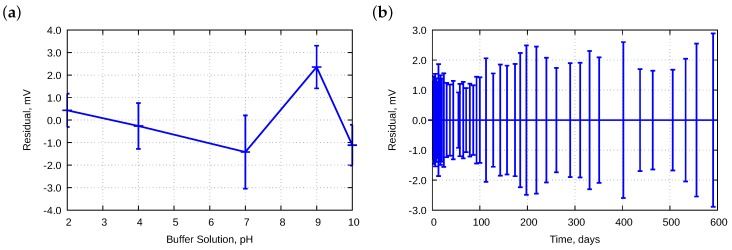
Averaged residuals obtained during approximation to the Nernst equation for electrodes E01–E11 as a function of: (**a**) pH; and (**b**) lifetime. Bars mean standard deviations.

Electrode parameters were approximated using the least squares regression. It was impossible to plot a straight line that goes through all five points obtained experimentally. Therefore, some residuals were produced. Figure 7 presents the residuals calculated for electrodes E01–E11. 

Due to the huge number of data, two cross-sections were performed. Figure 7a presents dependencies of pH. We observed some asymmetry; residuals at pH 9 were primarily positive and could be caused by a deviation of the buffer solution. The offset was approximately 2.5 mV which corresponds to approximately 0.042 pH. According to the manufacturer’s data, the pH was in ±0.05 pH range. Therefore, such an offset should be considered as acceptable. Figure 7b presents changes in the residuals in time. The best properties were obtained between Day 30 and Day 100—the residuals were within ±2.5 mV with standard deviation approximately 1.2 mV. With further time, the range was slightly widened.

**Figure 8:**
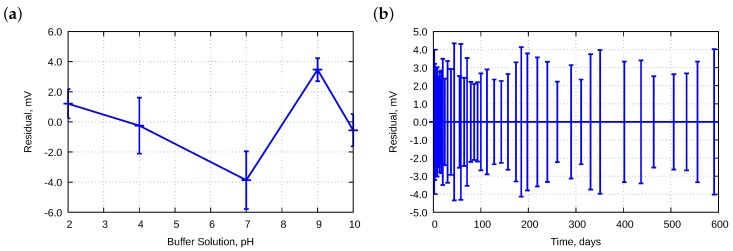
Averaged residuals obtained during approximation to the Nernst equation for electrode E12 as a function of: (**a**) pH; and (**b**) lifetime. Bars mean standard deviations.

Figure 8 presents the residuals for electrode E12.

Figure 8a is similar to that of Figure 7a. Differences are observed in Figure 8b and Figure 7b. The E12 electrode did not improve its performances between Day 30 and Day 100. It could be explained by the fact that the electrode was older, i.e. approximately two years old. In the meantime, the electrode was stored in a KCl solution.

**Figure 9:**
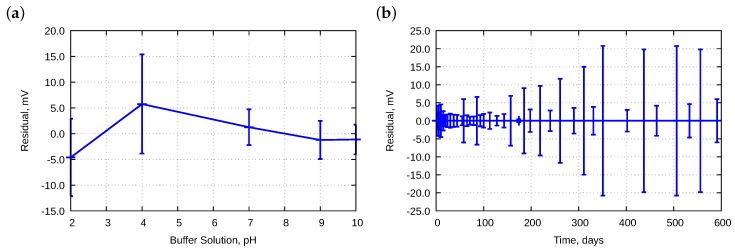
Averaged residuals obtained during approximation to the Nernst equation for electrode E13 as a function of: (**a**) pH; and (**b**) lifetime. Bars mean standard deviations.

The old electrode E13 fitted much worse to the Nernst equation. Its residuals are presented in Figure 9. They were nearly ten-fold larger. The hysteresis effect, which is described in Section 4.6, was also observed. 

### 4.6. Hysteresis

We carried out the experiments such that in the following days in which the measurements were performed, the electrodes were placed alternately in the buffer solutions beginning from pH 2 to pH 10 and vice-versa—from pH 10 to pH 2. Earlier sections note some influence of the order of measurements. For this reason, we decided to extend the analysis of this phenomenon and quantitatively determine its magnitude.

**Figure 10:**
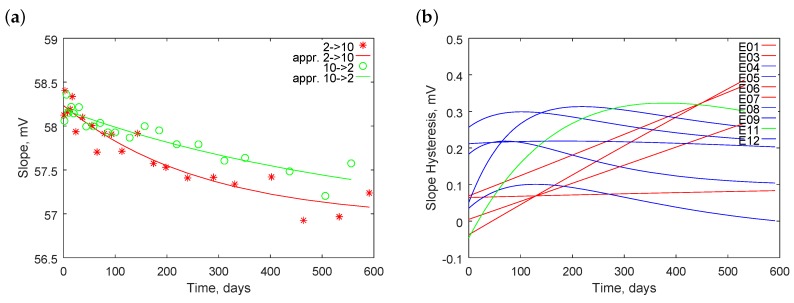
Illustration of dependence of hysteresis effect on electrode slope: (**a**) example of electrode E11; and (**b**) hysteresis of electrodes E01–E12.

For every electrode, we calculated two time-dependent approximation functions describing changes of the electrode slope: for increasing and decreasing pH, respectively. Because the exponential function better fit the parameter, it was used here as well. Plots for all the electrodes were performed, such as those presented in Figure 10a.

The difference between the two functions is a hysteresis and is graphically presented in Figure 10b. We recognized three types of time-dependencies. The first type, plotted in red, was characterized with a nearly linear increase with time. The second type, plotted in blue, had an increase in hysteresis at the initial time and, subsequently, its value decreased. In the third type, plotted in green, the hysteresis also increased with time, but the increase was faster initially than in the later stage.

The hysteresis is quantitatively determined (Table 6).

Except for the E13 electrode, the approximated hysteresis at the beginning of the tests were below 0.26 mV; up to 300 days, the values were below 0.31 mV; and up to 600 days they were below 0.45 mV. Due to the dynamic properties of the electrode, the hysteresis grew over time. The longer response was caused by the increasing thickness of the hydrated layer on the glass membrane [35]. The calculated slope hysteresis expressed in mV propagated to the measured pH value with the sensitivity coefficient proportional to the measured pH divided. This means that the absolute error of the pH measurement increased with pH, e.g., 0.45 mV at 10 pH corresponds to $0.41\;\mathrm{mV}\cdot 10\;\mathrm{pH}/59\;\frac{\mathrm{mV}}{\mathrm{pH}}=0.07$ pH. Electrode E13 had a hysteresis approximately 20-fold worse and therefore is not suitable for use.

**Figure 11:**
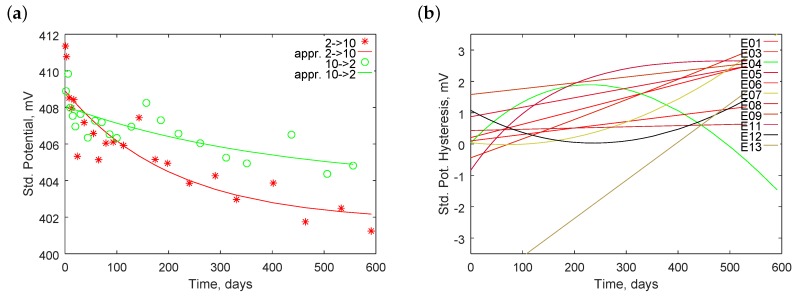
Illustration of dependence of hysteresis effect on electrode standard potential: (**a**) example of E11; and (**b**) hysteresis of all electrodes tested.

Similar tests were performed for the standard potential. Figure 11a presents an example of two functions, approximating data collected with the increase and decrease of pH, respectively.

Figure 11b plots the hysteresis of electrodes E01–E12. Most electrodes exhibited a linear increase in the hysteresis with time. Only electrodes E04 and E12 exhibited a different shape of dependence. Further analyses, not included in this paper, show the large contributions of random errors. Moreover, fitting to a function in the case of E13 was difficult due to random errors; therefore, the obtained line is untrustworthy.

Assessment of the standard potential hysteresis is included in the second part of Table 6. In the beginning, it was in the limits of 1.6 mV that correspond to 0.027 pH; within 300 days, it was within 2.4 mV $\hat{=}$ 0.041 pH; and within 600 days, it was appropriately 3.7 mV $\hat{=}$ 0.063 pH.

**Figure 12:**
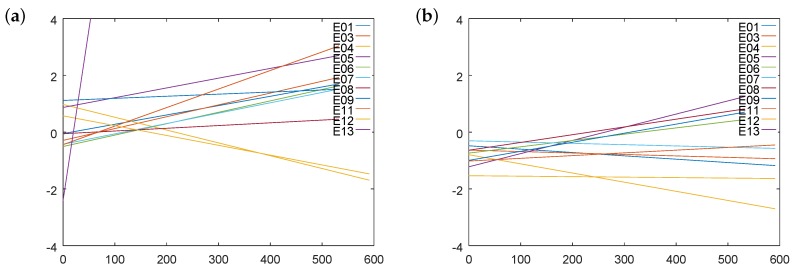
Illustration of dependence of hysteresis effect on electrode potential: (**a**) at 4 pH; and (**b**) at 9 pH.

Analogous calculations could be conducted to determine values for electrode potentials. Plots for pH 4 and 9 are presented in Figure 12.

Similar charts are plotted for the remaining buffer solutions. The hysteresis of electrode potential could be lower than that of standard potential, e.g., for E12. It resulted from the fact that standard potential and slope were correlated with each other; therefore, their contributions to electrode potential were partially compensated.

Values of approximated potential hysteresis are recorded in Table 7.

For analysis, the absolute values should be used. Moreover, electrode E13 exhibited the worst performance. A deeper analysis of electrode E12 showed weak fitting to the approximation lines due to random errors. Excluding the two electrodes at the beginning of the tests, all hysteresis were within 1.5 mV $\hat{=}$ 0.025 pH. It was a better result than that for the standard potential. Approximations of hysteresis at Day 300 are collected in the second part of the table. Here, electrode E13 significantly differed from others. The remaining electrodes provided absolute values within 2.2 mV $\hat{=}$ 0.037 pH. Approximations at Day 600 are recorded in the third part of the table. The worst case was within 4 mV $\hat{=}$ 0.068 pH.

The data presented above show that we cannot simply sum the hysteresis of the standard potential and slope to calculate the hysteresis for electrode potential and further for the measured pH. The approximations were conducted over other cross-sections. In the case of electrode parameters, the parameters were first approximated, i.e. averaged, based on five measurements. Subsequently, time changes were considered. In the case of electrode potentials, only time changes were observed without averaging over buffers. Therefore, the obtained results only provide few hints regarding the magnitudes.

## 5. Impact of Long-Term Changes on Measurement Uncertainty

Measurement uncertainty evaluation consists of several steps. In the first one, all uncertainty sources should be enumerated. In addition to the sources which are typical in laboratory tests, such as the repeatability of indications, the accuracy of buffer solutions, and the linearity of the Nernstian characteristic, the sources of uncertainty that have been described in this paper should also be considered, especially in long-term industrial applications. These are the changes of calibration parameters in the time between calibrations, the increase in linearity errors, and the increase in hysteresis.

Determination of the magnitudes of the uncertainty sources is a time-consuming task. It is based on observations and is difficult to generalize; even electrodes of the same type have different long-term properties. An assumption of limiting values could lead to an unjustified increase in the estimated uncertainty of the measured pH value.

When uncertainty values of the sources are already known, their contribution to the combined uncertainty should be described. To do this, the sensitivity coefficients should be calculated. Because the slope and the standard potential are correlated with each other, the task becomes even more complex. Presentations of numerical values for the investigated electrodes significantly exceed the scope of the paper and, therefore, are omitted.

The last step includes the presentation of the measurement results in the way recommended by international standards. In addition to the measured value, it should contain a 95% confidence interval. Doubled combined uncertainty is nearly always treated as the interval.

## 6. Conclusions

This work describes a nearly 600-day aging process of eleven potentiometric glass pH electrodes. During this study, we measured the potentials of all the electrodes in five buffer solutions 44 times.

Between the measurement series, the electrodes were stored in tap water. The time intervals between the series were different. We observed various drifts in the potentials. The drifts were more linear as a function of time than as a function of the measurement number. This means that the aging process was dependent predominantly on time and not on electrode exploitation. Considering each of the electrodes separately, the potentials in each of the five buffer solutions drifted at a different rate.

We calculated the parameters of the Nernst model for each electrode after each measurement series. The parameters also drifted. For most electrodes and storing in this particular tap water, a linear model describing time-dependency was adequate, but some electrodes were in better accordance with exponential functions. The developed aging models in other mediums might be significantly different. The steady states and time constants of the exponential functions were difficult to justify theoretically. Because a correlation exists between the electrode slope and the standard potential, it was possible to calculate time-independent pH values. The obtained values for the investigated electrodes were, however, out of the usual pH range and hence useless.

We tested the predictions of the electrode parameters based on their past parameters and the obtained aging model. The predicted parameters allowed for the improvement of measurement accuracy between calibrations on-line in the time of measurements, and not only as post-processing. Too long predictions increased errors.

Observations of linearity of electrode responses led to the following conclusion. The electrodes performed the best between Day 30 and Day 100 of its exploitation. The memory effect, its hysteresis, also changed with time. Usually, the hysteresis increased in time due to the changes in electrode dynamic properties. The hysteresis of the standard potential usually changed linearly, whereas that of the electrode slope changed exponentially.

During assessments of uncertainties in industrial measurements performed using pH electrodes, three additional factors should be taken into consideration: drifts of electrode parameters, hysteresis, and linearity. In usual laboratory tests, the factors have much less impact.

## Figures and Tables

**Table 1:** Electrodes used in investigations. All electrodes are of 12-mm diameter.

Symbol	Type	Age	Shape	Liquid Junction
E01	ERH-11	new	spherical	ceramic
E03	ERH-11	new	spherical	ceramic
E04	ERH-11A	new	spherical	triple, ceramic
E05	ERH-11S	new	spherical	glass, sleeve
E06	ERH-11X	new	spherical	ceramic
E07	ERH-12	new	conical	ceramic
E08	ERH-13	new	cylindrical	ceramic
E09	ERH-111	new	cylindrical	polyester fiber
E11	ERH-11	1 y.o.	spherical	ceramic
E12	EPX-3	2 y.o.	flat	Teflon o-ring
E13	ERH-11	old	sphere	ceramic

**Table 2:** Drift parameters of electrode slope approximated by linear or exponential functions. $S_{0}$, initial value; ΔS, daily change; SSR, sum of squares of residuals; $S_{\infty }$, final value; $T_{S}$, time constant; $\Delta S_{0}$, initial daily change.

	Linear			Exponential				
Electrode	$S_{0}$	ΔS	SSR	$S_{0}$	$S_{\infty }$	$T_{S}$	$\Delta S_{0}$	SSR
	mV	μVday	${\mathrm{mV}}^{2}$	mV	mV	Day	μVday	${\mathrm{mV}}^{2}$
E01	58.28	−2.3	1.02	58.28	−70,416.19	30,727,308	−2.3	1.02
E03	58.39	−3.1	1.49	58.39	−84,460.73	27,464,834	−3.1	1.49
E04	58.13	−1.6	2.64	58.32	57.41	149	−6.1	1.80
E05	58.06	−1.7	3.68	58.30	57.35	116	−8.2	2.59
E06	58.31	−2.1	0.47	58.31	−62,932.20	29,702,315	−2.1	0.47
E07	58.31	−2.4	0.74	58.31	−52,209.03	21,803,321	−2.4	0.75
E08	58.20	−1.2	1.60	58.33	57.64	163	−4.2	1.04
E09	58.19	−1.1	1.61	58.30	57.70	155	−3.9	1.40
E11	58.15	−2.0	1.24	58.19	56.14	745	−2.8	1.19
E12	58.13	−2.8	2.86	58.22	55.98	481	−4.7	2.55
E13	54.80	−5.2	323	54.80	−10,372,169.41	1,998,707,810	−5.2	3

**Table 3:** Drift parameters of electrode standard potential approximated using linear or exponential equations. $E_{0}^{\circ }$, initial value; $\Delta E^{\circ }$, daily change; SSR, the sum of squares of residuals; $E_{\infty }^{\circ }$, final value; $T_{E^{\circ }}$, time constant; $\Delta E_{0}^{\circ }$, initial daily change.

	Linear			Exponential				
Electrode	$E_{0}^{\circ }$	$\Delta E^{\circ }$	SSR	$E_{0}^{\circ }$	$E_{\infty }^{\circ }$	*T*	$\Delta E_{0}^{\circ }$	SSR
	mV	μVday	${\mathrm{mV}}^{2}$	mV	mV	day	μVday	${\mathrm{mV}}^{2}$
E01	417.36	−46	63.1	417.36	−669,941,761,662.4	14,433,838,818,136	−46	63.1
E03	414.60	−45	69.7	414.6	−19,874,060.63	443,190,819	−45	69.7
E04	411.86	−164	227.8	413.9	172.33	1194	−202	89.0
E05	422.30	−46	107.9	422.3	−1,462,695.47	31,651,419	−46	107.9
E06	406.53	−53	114.5	406.53	−42,535,985.41	797,369,242	−53	114.5
E07	411.98	−86	35.3	412.04	−1181.02	18263	−87	35.1
E08	426.39	−184	206.4	426.39	−2,554,655.69	13,882,511	−184	206.5
E09	410.18	−11	124.0	410.18	−968,890.46	89,136,112	−11	124.0
E11	408.08	−11	100.5	408.13	376.24	2675	−12	100.4
E12	414.98	−50	259.9	415.26	270.48	2648	−55	256.8
E13	391.41	49	5693	382.62	413.27	112	274	3935

**Table 4:** Values of pH for which the electrode potential is independent of its lifetime.

Electrode	E01	E02	E04	E05	E06	E07	E08	E09	E11	E12	E13
${\mathrm{pH}}_{\mathrm{indep}.}$	20.22	14.52	103.76	27.84	25.13	35.87	168.22	9.89	5.48	17.52	−9.46

**Table 5:** Quality factors of predictions calculated for slopes and standard potentials. Off-line factors are standard deviations of differences between parameters obtained from calibration and from exponential approximation. On-line factors are standard deviations of differences between parameters obtained from calibration and from the prediction of future *n* values, where *n* is the number of future predictions. All values are expressed in mV.

		On-Line						On-Line				
Electrode	Off-Line	n=1	n=2	n=3	n=4	n=5	Off-Line	n=1	n=2	n=3	n=4	n=5
	For Slopes						For Standard Potentials					
E01	0.16	0.25	0.23	0.24	0.18	0.28	1.2	1.7	1.8	1.8	1.7	2.1
E03	0.19	0.26	0.28	0.28	0.29	0.37	1.3	1.8	1.8	2.2	2.2	2.7
E04	0.21	0.24	0.21	0.26	0.21	0.29	1.5	1.8	1.8	2.2	2.2	3.0
E05	0.25	0.46	0.53	0.51	0.59	0.66	1.6	1.7	1.8	2.1	2.2	2.4
E06	0.11	0.13	0.13	0.14	0.14	0.16	1.7	2.0	2.3	2.7	3.1	3.4
E07	0.13	0.17	0.17	0.18	0.17	0.18	0.9	1.4	1.5	1.8	1.9	2.1
E08	0.16	0.22	0.26	0.30	0.35	0.39	2.2	2.0	2.1	2.2	2.1	2.2
E09	0.18	0.23	0.24	0.27	0.26	0.26	1.7	1.8	1.9	2.1	2.0	2.0
E11	0.16	0.21	0.24	0.25	0.29	0.29	1.3	1.5	1.4	1.7	1.6	1.8
E12	0.25	0.52	0.53	0.44	0.71	0.66	2.5	3.5	3.9	4.1	4.4	4.4
E13	2.77	3.27	2.93	3.32	2.92	3.40	14.5	12.9	12.7	16.2	16.8	19.2

**Table 6:** Approximated values of the hysteresis on the indicated days (in mV).

Electrode	E01	E02	E04	E05	E06	E07	E08	E09	E11	E12	E13
slope											
day 0	0.07	−0.04	0.05	0.18	0.06	0.01	0.04	0.26	−0.04	0.21	−1.4
day 300	0.24	0.21	0.30	0.15	0.07	0.15	0.07	0.26	0.31	0.22	−5.1
day 600	0.41	0.44	0.25	0.10	0.08	0.30	0.00	0.22	0.28	0.20	−8.8
time-dependence type	1	1	2	2	1	1	2	2	3	2	1
standard potential											
day 0	0.20	−0.44	0.03	0.87	0.10	0.04	0.43	1.58	−0.83	1.07	−4.80
day 300	1.49	1.41	1.74	1.78	0.71	0.70	0.55	2.14	2.40	0.10	−1.16
day 600	2.78	3.40	−1.61	2.69	1.32	3.62	0.66	2.69	2.63	2.10	2.49

**Table 7:** Approximated values of the electrode potential hysteresis (in mV) in buffer solutions.

Electrode	E01	E02	E04	E05	E06	E07	E08	E09	E11	E12	E13
at the beginning of experiments											
2 pH	0.17	−0.08	−0.43	−0.07	0.46	0.12	0.62	0.49	−1.11	0.26	0.66
4 pH	−0.05	−0.29	0.97	0.87	−0.51	−0.43	−0.06	1.11	−0.44	0.57	−2.41
7 pH	−0.56	−0.87	−0.52	−0.80	−0.89	−0.84	−0.92	−0.25	−0.79	−2.90	4.14
9 pH	−0.48	−0.63	−0.80	−1.22	−0.74	−0.31	−0.64	−1.00	−1.02	−1.54	6.32
10 pH	−0.24	0.73	−0.36	−1.13	0.10	−0.09	0.16	−1.41	0.09	−0.44	11.1
at day 300											
2 pH	0.78	0.82	0.80	1.02	0.47	0.46	0.44	1.31	0.98	0.79	−1.84
4 pH	0.93	0.96	−0.38	1.90	0.70	0.67	0.23	1.34	1.52	−0.47	33.7
7 pH	−0.31	−0.04	−0.97	0.92	−0.07	0.03	0.84	0.77	0.37	−1.57	35.8
9 pH	−0.84	−0.79	−1.76	0.17	−0.07	−0.44	0.18	−0.04	−0.73	−1.59	40.3
10 pH	−0.76	−0.31	−2.17	0.53	0.16	−0.32	0.03	−0.77	−0.35	−0.44	49.1
at day 600											
2 pH	1.40	1.86	−2.39	2.12	0.46	2.10	0.27	2.14	0.98	1.36	−4.35
4 pH	1.92	2.21	−1.73	2.93	1.90	1.76	0.52	1.56	3.47	−1.50	69.9
7 pH	−0.06	0.79	−1.43	2.64	0.75	0.89	2.59	1.79	1.52	−0.25	67.5
9 pH	−1.19	−0.94	−2.73	1.56	0.59	−0.58	1.00	0.92	−0.44	−1.64	74.3
10 pH	−1.27	−1.34	−3.98	2.19	0.23	−0.54	−0.10	−0.13	−0.79	−0.44	87.1
